# Non-Destructive Detection of Wire Rope Discontinuities from Residual Magnetic Field Images Using the Hilbert-Huang Transform and Compressed Sensing

**DOI:** 10.3390/s17030608

**Published:** 2017-03-16

**Authors:** Juwei Zhang, Xiaojiang Tan, Pengbo Zheng

**Affiliations:** 1 College of Electrical Engineering, Henan University of Science and Technology, Luoyang 471023, China; 13598163915@163.com; 2 Power Electronics Device and System Engineering Laboratory of Henan, Henan University of Science and Technology, Luoyang 471023, China

**Keywords:** wire rope, residual magnetic field, Hilbert-Huang transform, compressed sensing, quantitative recognition

## Abstract

Electromagnetic methods are commonly employed to detect wire rope discontinuities. However, determining the residual strength of wire rope based on the quantitative recognition of discontinuities remains problematic. We have designed a prototype device based on the residual magnetic field (RMF) of ferromagnetic materials, which overcomes the disadvantages associated with in-service inspections, such as large volume, inconvenient operation, low precision, and poor portability by providing a relatively small and lightweight device with improved detection precision. A novel filtering system consisting of the Hilbert-Huang transform and compressed sensing wavelet filtering is presented. Digital image processing was applied to achieve the localization and segmentation of defect RMF images. The statistical texture and invariant moment characteristics of the defect images were extracted as the input of a radial basis function neural network. Experimental results show that the RMF device can detect defects in various types of wire rope and prolong the service life of test equipment by reducing the friction between the detection device and the wire rope by accommodating a high lift-off distance.

## 1. Introduction

Wire ropes are widely employed components in diverse areas, such as in industrial production, tourist cable cars, mining, metallurgy, shipbuilding, and elevators. Wire rope is a heavily loaded component, and long-term continuous operation eventually result in corrosion, wear, broken wires, loose wires, and fatigue, which decrease the loading strength of the rope, and can cause accidents, resulting in property damage and injury [1]. The traditional damage detection method is artificial visual inspection, which is a low efficiency, time-consuming, and unreliable method [1]. The development of a fast, non-destructive, and automatic detection technology is therefore necessary.

Wire rope defects include three main types: the loss of metallic area (LMA), local faults (FLs), and structural faults (SFs). The main non-destructive testing (NDT) methods employed for wire rope inspection include electromagnetic detection, ultrasonic guided wave (UGW) evaluation, radiation testing, eddy current inspection, and optical detection [1]. However, designing a precise detection device that can quantitatively determine the characteristics of defects, such as the number of broken wires, remains problematic, particularly when operating in severe environments [2].

The UGW method has been shown to provide a detection speed that is faster than other methods, but the method demonstrates a low anti-interference ability and suffers from strong background noise [3,4,5,6,7]. Treyssède and Laguerre’s [3] applied the transmission characteristics of UGW for wire rope testing. The researchers developed a semi-analytical finite element method, and calculated the optimal excitation and receiving sites. This approach provided a wave dispersion curve for spiral steel rope. Vanniamparambil [4] proposed a novel detection method that combined three technologies: UGW, acoustic emission techniques, and digital image processing. Xu [7] evaluated the detection precision of the UGW method for wire rope defects obtained at different frequencies, showing that wire ropes at higher frequencies had longer recovery lengths for their elastic waves. Raisuitis [5] investigated the propagation of UGWs along composite multi-wire ropes with various types of acoustic contacts between neighboring wires and the plastic core. Tse and Rostami [6] investigated the efficiency of employing the magnetostriction of ferromagnetic materials in conjunction with the UGW method for wire rope defect inspection, and the location and severity of defects were approximately identified and characterized using the short-time Fourier transform and wavelet analysis. Other detection methods, such as radiation testing [8] and eddy current inspection [9], have not been applied to wire rope inspection to a large extent.

**Figure 1:**
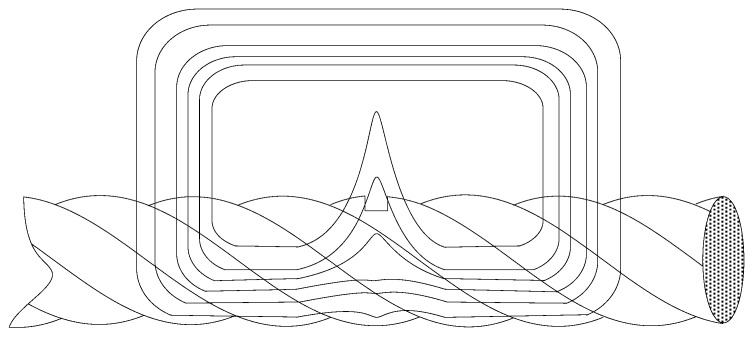
Magnetic circuit of a defect in a wire rope.

Electromagnetic detection methods are commonly employed for the NDT of wire rope [2]. The basic principle behind wire rope electromagnetic detection is illustrated in Figure 1. The lower permeability of the air leads to magnetic field leakage (MFL) from the rope defect, and the strength of the MFL can be obtained from an appropriately designed magnetic detection device. In terms of the type of excitation source employed, electromagnetic detection can be divided according to the use of a coil [10,11] or a permanent magnet [12,13,14,15,16,17] for generating a magnetic field. Modified main-flux equipment has been developed for wire rope inspection, which induced changes in the electromagnetic field strength owing to the leakage field derived from defects in various large-diameter wire ropes [10]. Other researchers [11] employed a pair of saddle coils for the magnetization of a steel track rope, and this system was applied to detect small, inner flaws in the rope. Permanent magnets have been employed in a saddle structure to saturate wire rope in a uniform magnetic field [14,15,16,17]. Wang et al. [12] investigated the effect of excitation distance and the lift-off distance between the sensors and the wire rope surface on the detection precision. The researchers accordingly modified the magnetic circuit of the detector to restrain the impact of fluctuations in the sensor lift-off distance. Xu et al. [18] developed a magnetic excitation model. Based on this model, the researchers established design criteria for the size of the excitation structure, proposed a theoretical framework for the excitation structure size based on numerical analysis, and adjusted the theoretical design using finite element analysis (FEA).

Obtaining a precise MFL signal is the most important aspect for the accurate electromagnetic NDT of wire rope. For MFL signal acquisition, a commonly employed in-service NDT method utilizes an induction coil [10,17], Hall effect sensor [14,18,19,20,21], giant magnetoresistive (GMR) sensor [11,22], and tunnel magnetoresistive (TMR) sensor [23]. Jomdecha and Prateepasen [10] modified a conventional induction coil into a coil array that densely covered the wire rope to acquire the MFL signal. Wang and Tian [14] utilized FEA to address the problems associated with the weak MFL signals derived from small defects, and they investigated the gathered magnetism of the magnetization rope. They designed a detector with an annular pole polymagnet on one side using Hall elements as inductors. This detection system was able to capture weak MFL signals within the strong magnetic field. Xu and Wang [18] developed an online modular-detector NDT system using a Hall effect sensor that successfully detected inner broken wires. The researchers also presented three filtering algorithms. Detectors based on Hall effect sensor arrays have been widely applied for NDT under strong magnetic field conditions [19,20,21]. Cao [19] created an image from the defect data which was obtained by Hall sensors array, and applied digital image processing to extract and detect defect characteristics. Zhang et al. [20] employed signal processing to suppress the effect of lift-off distance, and applied statistical processing to distinguish different types of defects and to obtain binary image data describing the spatial extent of defects. Zhang et al. [21] applied a space filter to suppress the texture of strand waves after obtaining MFL gray-level images of wire rope defects, and the image spectrum texture was extracted as the characteristic vector used for recognition. GMR sensors have been employed for MFL signal acquisition because of their high sensitivity, high precision, and small size. GMR sensors were placed into a sensor array and densely distributed on the wire rope surface in a manner similar to that employed in a Hall effect sensor application [11]. Zhang and Tan [22] utilized the high sensitivity of a GMR sensor to develop a detection technique based on remanence magnetization, which combined the benefits of a simple structure and high detection speed with high precision. Wu et al. [23] demonstrated that TMR sensors can be applied to detect small discontinuities on a wire rope surface.

MFL signals contain a variety of distinct noise signals, which makes the development of an efficient de-noising algorithm challenging work. Currently, a number of noise reduction algorithms are commonly employed, including wavelet analysis de-noising, low-pass filter, notch filter [21], adaptive filter [20], morphological filter [24], and a de-noising algorithm based on compressed sensing (CS) [22]. Zhang et al. [20] applied digital image processing to develop a space filter for smoothing the defects in an MFL signal image. Zhang et al. [21] proposed a baseline estimation algorithm to suppress the effect of undulations in the lift-off distance and an adaptive notch filtering algorithm to filter the strand wave for increasing the signal-to-noise ratio. Zhang and Tan [22] utilized wavelet multi-resolution analysis to eliminate the baseline of the signal. Their work was based on the CS wavelet de-noising algorithm, and they calculated the best sparse transform expression to completely filter out the noise. Tian et al. combined the wavelet transform and the morphological transform to create a morphological filter algorithm that suppressed the interference associated with the baseline drift in the wire rope signal. Artificial neural networks have been widely applied to realize the quantitative detection of wire rope defects. These networks operate much like back propagation (BP) neural networks employed by a number of researchers [20,21,22]. However, BP neural networks suffer from some limitations and shortcomings, such as poor generalization and slow convergence.

Devices based-on the x-ray method are generally bulky, heavy, and suffer from high maintenance costs, and furthermore, it is difficult to accurately locate defect positions with the UGW detection method, which suffers from external disturbances and limited detection length. The eddy current inspection method is difficult to apply in practice for producing coils on-site, and the system is difficult to manage. However, conventional electromagnetic NDT typically utilizes a large, heavy excitation device as well. Moreover, extracting defect information from the induction coil is difficult, and, in addition, the sensitivity of Hall effect sensors is low. Conventional digital filter algorithms cannot adequately suppress the noise in the MFL signal. This limited ability to reduce noise makes it difficult to separate the defect signal and the strand wave under low signal-to-noise ratio conditions while processing the signal.

To overcome the disadvantages of existing detection devices, we developed a prototype device based on the RMF of a wire rope. This inspection method utilizes GMR sensors for excitation signal acquisition. After magnetizing the wire rope with permanent magnets, the GMR sensor array was utilized to obtain the RMF strength of the rope surface. This detection system is non-contact and non-invasive which prolongs the service life of test equipment. A novel filter algorithm based on the Hilbert-Huang transform (HHT) and compressed sensing wavelet filtering (CSWF) was developed to suppress the various system noises. The HHT was employed to remove the DC component of the signal and balance the sensor channels. CSWF was employed to suppress high-frequency noises and strand waves. Then, we applied digital image processing to create a binary image using a filter based on corrosion and expansion. Subsequently, defects were located and segmented within the gray-level image. Because an 18 GMR sensor array was employed, the resulting gray-level image included only 18 pixels in its circumference. Three spline interpolations were performed to improve the circumferential resolution of the gray-level image. Thirteen image characteristics comprising 6 image textures and seven invariant moments were extracted as defect feature vectors. A radial basis function (RBF) neural network, which is a fast-learning classification network that provides a global optimum, was adopted to quantitatively detect the number of broken wires in the rope. Experimental results demonstrate that, when the absolute limiting error for the detected number of broken wires is 2, the recognition rate is as high as 93.75% with an average recognition error of 0.7813 wires.

## 2. RMF Detection

### 2.1. Platform Design

**Figure 2:**
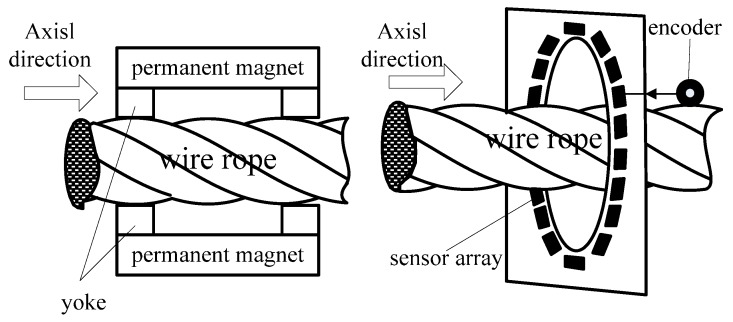
Schematic diagram of the RMF detection process.

Wire rope consists of flexible ferromagnetic steel wires twisted in a helix structure around a hemp core. Based on quantum mechanics, the exchange coupling of an electron spin magnetic moment produces magnetic domains in ferromagnetic materials [25]. When there is not magnetisation, a ferromagnetic component produces little external magnetic field. However, when the nuclear charge is distributed consistently, saying that wire ropes magnetise, the component generates a magnetic field, namely the RMF. Based on this property, we designed a prototype device for the detection of wire rope defects. The detection principle is illustrated in Figure 2. First, a device consisting of permanent magnets as an excitation source was used to completely homogenize the magnetisation of wire rope. Then, a GMR sensor array and an ARM controller board were applied to obtain the RMF distribution of the wire rope.

**Figure 3:**
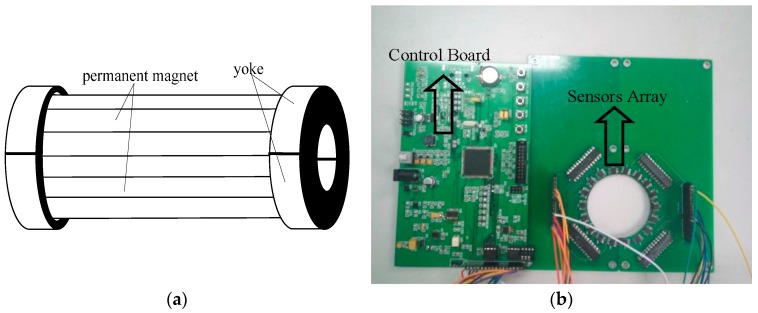
(**a**) Schematic diagram of the excitation device; (**b**) The printed circuit boards employed in the detection system, including the control board and a sensor array board.

The excitation source of the device was constructed of small magnets placed end-to-end to form a magnetic strip. Two half-ring magnetic yokes held the strips and formed a semi-cylinder. The two semi-cylinders created a complete cylinder that was arranged on the surface of the rope. A schematic diagram of the excitation device and an image of the printed circuit boards of the system are shown in Figure 3. The magnetic sensors were AAH002 analog GMR sensors (NVE Corp., Minneapolis, MN, USA), and 18 GMR sensors were employed in the sensor array. The system control board consisted of an encoding module, signal amplifier module, array control module, data storage module, and CPU module. We employed an STM32F407 CPU core because it incorporates a 12-bit analog-to-digital converter (ADC).

### 2.2. Data Acquisition

**Figure 4:**
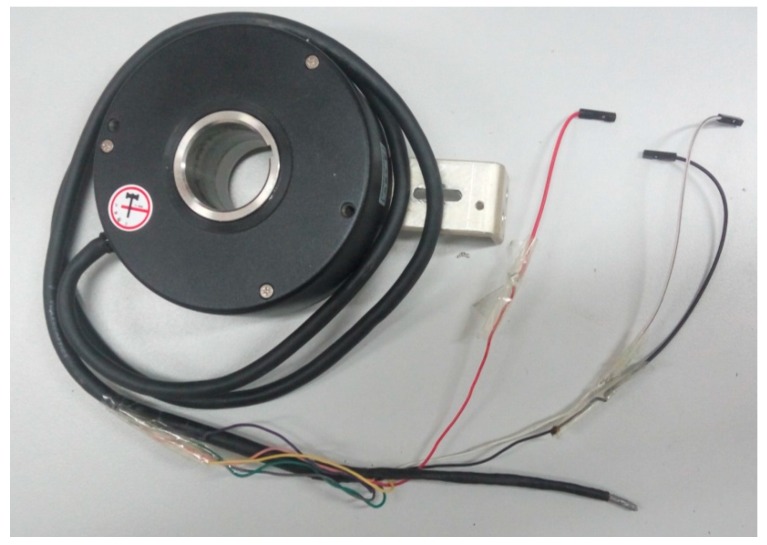
Image of the encoder.

The magnetic sensitivity of the GMR sensors ranged linearly over 0.6–3 Oe (Gauss in air), and the 18 sensors were densely distributed around the wire rope. The ARM9 core controlled all system operations, and a TF card was used to store the raw RMF data. The sampling pulses were produced by the encoder shown in Figure 4). Equal-spaced sampling was implemented on the wire rope (1024 pulses while the inner circle of encoder rotated 0.35 m).

After the magnetisation of wire rope was completely homogenized by the magnetization device, a weak magnetic field existed on the surface of the rope, which was stable over a short period (the data acquisition operation could be implemented over the following week without magnetizing the system again). In the experiments, artificial discontinuities (i.e., broken wires) were introduced into four types of wire rope: 6 × 19, 6 × 36, 6 × 27, and 7 × 27 (strands × wires per strand). While the diameter of the wires ranged from 22 to 36 mm, we primarily focused on ropes 30 mm in diameter because they produced an optimal lift-off distance when acquiring data. For every encoder pulse signal, the signals obtained from 18 GMR sensors were transformed into a digital signal by the ADC.

### 2.3. RMF Image

**Figure 5:**
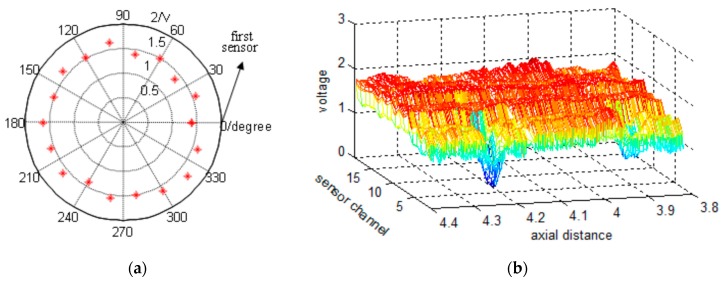
(**a**) The 18 circumferential data points obtained from GMR sensors for a single pulse plotted in polar coordinates (the radius represents the voltage); (**b**) Partial raw data expanded image.

We collected RMF data from each of the GMR sensors with every pulse. Figure 5a shows the 18 data points obtained for a single pulse plotted in polar coordinates in intervals of 20°, where the radius represents the sensor voltage. After completing equal-spaced sampling, an M × N data matrix was obtained, where M is the number of sensor channels and N is the number of pulses received from the encoder. The data matrix was then expanded into two-dimensional images from first sensor channel. Figure 5b shows a segment of raw data expanded in the axial direction from the first sensor channel to the eighteenth.

## 3. Signal Processing

**Figure 6:**
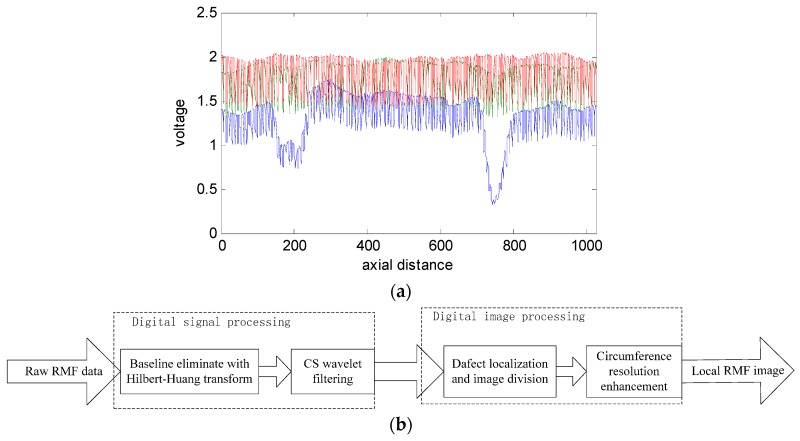
(**a**) One-dimensional waveforms of three channels of raw data (the data is derived from Figure 4b; (**b**) Data processing flow diagram.

The signal-to-noise ratio of the raw data shown in Figure 5b is obviously low. The observed noise may be produced by the helix structure of the wire rope, magnetic leakage between the steel wires, uneven magnetization, or an unbalanced sensor channel caused by various lift-off distances. Three channels of raw data are plotted as a one-dimensional waveform in Figure 6a. It is obvious that the aforementioned noise sources make it difficult to locate and extract the defects. Moreover, a considerable degree of imbalance between channels is observed in Figure 6a. Hence, implementing filtering operations is necessary. The data processing flow diagram is shown in Figure 6b.

As shown in Figure 6b, the digital signal processing flow consists of two components, where one provides reprocessing for removing the DC component of the signal and suppressing low and high frequency noise signals, which ensures a balanced RMF signal. The HHT is more adaptive than wavelet analysis because an appropriate wavelet basis is often difficult to find. In addition, the sparsity of defects obtained in the RMF signal is ideally suited to CSWF [22].

### 3.1. Reprocessing Theory

The HHT [26] is a novel adaptive time-frequency analysis technique that consists of two components: empirical mode decomposition (EMD) and the Hilbert transform. The EMD algorithm decomposes the signal into a series of intrinsic mode functions (IMF). Then, the Hilbert transform is implemented to calculate the instantaneous frequency of the IMFs. The instantaneous frequency, illusive components, direct current component, and highest frequency components in the IMFs were eliminated to enhance the signal-to-noise ratio, while the residual components were added together. Based on EMD theory, the IMF components must satisfy the following basic conditions [26].

1. The average number of maxima and minima of an IMF component must be equivalent to the number of 0 crossings, or they differ by 1 at most.
2. The average of the maxima and minima, as defined by the envelope, should be 0 at any given moment.

However, when implementing the EMD algorithm, modal mixing and boundary effects will occur between the IMFs. The theory indicates that the average of repeatedly decomposing IMFs adds Gaussian white noise to the residual signal, which can suppress the modal mixing phenomenon. Utilizing cubic spline interpolation results in undulating fitting errors in the signal boundary, and repeatedly decomposing the residual signal leads to the propagation of these errors, which is the cause of the boundary effect. The mirror extending method has been shown to be effective for suppressing this negative effect [27]. This improved decomposition algorithm, denoted as ensemble EMD (EEMD), is described as follows [28]:

(1) First, extend the raw signal to obtain $\tilde{x}(t)$, and initialize the residual signal $r_{n}$, IMFs set $c_{i}$ as $c_{i}=\emptyset ,r_{n}=\tilde{x}(t)$.
(2) Add Gaussian white noise w(t) to $r_{n}$:
  (1) $$y(t)=\tilde{x}(t)+w(t).$$
(3) Implement EMD for y(t) to obtain IMF $c_{ij}$:
  (2) $$x(t)=\sum_{i=1}^{n}c_{ij}+r_{n}.$$
(4) Repeat steps (2) and (3) *k* times, obtaining an IMF set $c_{ij}(i\leq n,j\leq k)$, and calculate the average of the IMFs. Update $r_{n}$ as follows:
  (3) $$\{\begin{array}{c} c_{i}(t)=\frac{1}{k}\sum_{j=1}^{k}c_{ij}\\ r_{n}=r_{n-1}-c_{ij} \end{array}$$
(5) If *i* > *n* or $r_{n}$ cannot be further decomposed, the decomposition is complete. Otherwise, *i* = *i* + 1, and return to step (2).

Equation (2) indicates that the EMD algorithm is complete and error-free because the raw data can be exactly recovered by summing the IMFs. This decomposition algorithm is adaptive and locally orthogonal [27].

After decomposing the signal, the Hilbert transform is then implemented for all IMFs. The Hilbert transform is described as follows:

(4) $$H[c_{i}(t)]=\frac{1}{\pi }\int_{-\infty }^{\infty }\frac{c_{i}(\tau )}{t-\tau }dt.$$

We then construct an analytic function $z_{i}(t)$:

(5) $$z_{i}(t)=c_{i}(t)+jH[c_{i}(t)]=a_{i}e^{j\theta_{i}(t)}.$$

The amplitude and phase are separately expressed as follows:

(6) $$\{\begin{array}{c} a_{i}(t)=\sqrt{c_{i}^{2}(t)+H^{2}[c_{i}(t)]}\\ \theta_{i}(t)=\arctan(\frac{H[c_{i}(t)]}{c_{i}(t)}) \end{array}$$

Thus, the instantaneous frequency is defined as:

(7) $$\omega_{i}(t)=\frac{d\theta (t)}{dt}.$$

### 3.2. Compressed Sensing Theory

Compressed sensing (CS) theory indicates that a sparse signal (or a signal sparse in the transform domain) can be reconstructed with a linear and non-adaptive sampling frequency that is much lower than the Nyquist frequency. This signal processing method can be divided into three components: sparse signal decomposition, compressed sampling, and signal reconstruction. Assuming a known measurement matrix Φ ∈ *R^M×N^* *(M* ≪ *N)* and a noisy signal $x\in R^{N}$ (which is not a sparse signal), the signal cannot be reconstructed using CS theory. However, if the signal is sparse in the transform domain, it can still be reconstructed into the raw signal $\hat{x}$ [29]. Thus, we assume that a transformation basis $\psi \in R^{N\times N}$ exists, where, under this basis, the transformation of x is sparse, and the transformation coefficient of the noise is much smaller than that of the actual signal. The transformation is:

(8) ω=ψx,

and the linear measure y in matrix Φ is:

(9) y=Φω=Φ(ψx).

Now, consider that the transformation expression of the optimal signal $\hat{\omega }$ is reconstructed by the linear measure y. We then implement the inverse transform to obtain the clean signal $\hat{x}$. It is easy to show that the dimension of y is much less than that of $\hat{\omega }$; thus, the solution of Equation (9) is infinite or unsolvable. Assuming solutions of this equation exist, $\hat{\omega }$ can be accurately reconstructed by solving the following optimal 0-norm of y [30]:

(10) $$\begin{array}{l} \hat{\omega }=\arg\min{\left\|\omega \right\|}_{0}\;s.t.\;y=\Phi \omega \\ or:\hat{\omega }=\arg\min{\left\|y-\Phi \omega \right\|}_{2}^{2}+\lambda {\left\|\omega \right\|}_{0} \end{array}$$

The solution to this formula is unique in the statistical sense, and the average of the solution is the same as the expression in the transform domain. In addition, utilizing the measure matrix Φ should satisfy the uniform uncertainty principle (UUP) [31]. This means that, for a vector *S* of sparsity *K*, *S* meets the conditions:

(11) $$0.8\frac{M}{N}{\left\|S\right\|}_{2}^{2}\leq {\left\|\Phi S\right\|}_{2}^{2}\leq 1.2\frac{M}{N}{\left\|S\right\|}_{2}^{2}.$$

The matrix $\Phi \in R^{M\times N}$ meets the UUP for a set size *K*, where K≤MlogN. Common measure matrixes Φ are random matrixes, such as the Gaussian matrix, Bernoulli random measurements, Fourier measurement matrix, and non-correlation measurement matrix. The core of the filtering algorithm based on CS theory is the recovery of the sparse expression $\hat{\omega }$. However, calculating its 0-norm for Equation (9) is a difficult non-deterministic polynomial problem. A number of sub-optimal solution algorithms have been proposed by scholars, and these methods can be divided into two categories. The first category includes the convex optimization algorithms, which convert this problem into a calculation of the minimum 1-norm. The other category includes greedy algorithms, such as the orthogonal matching pursuit (OMP) algorithm [29]. Although the recovery accuracy of the OMP is poor, it is usually adopted in practice because it provides for rapid computation. The OMP algorithm was therefore implemented in the present study to obtain the sub-optimal solution of Equation (9) and a Gaussian random matrix was adopted as the measure matrix. The core of the algorithm is the closest matching column, which has the maximum inner product with the measurement residue, and is selected in a greedy fashion. Then, the least squares method is implemented to acquire the approximate sparse solution and update the residual measurement. When the number of iterations reaches the sparsity *K*, the selection process stops. Thus, the defect RMF signal can be extracted with the sparsity *K*.

### 3.3. Description of the De-Noising Algorithm

The HHT cannot completely separate the defect signal from the raw data. On the other hand, CSWF cannot separate the direct current component. Therefore, we propose a filtering algorithm based on both HHT and CSWF to de-noise the 18 channel RMF signal [22]. This procedure is described as follows:

i The EEMD described in Section 3.1 is applied to the raw data, and the reprocessing signal $\hat{X}$ is obtained.
ii Apply CSWF to the re-processed signal of the *i*-th channel:
  (1) The Mallat decomposition algorithm is applied, and the sparse expression $W_{j}$ of signal ${\hat{x}}_{i}$ is obtained for each scale j.
  (2) Randomly generate a Gaussian matrix Φ and calculate the linear measure under the matrix Φ: $y_{j}=\Phi W_{j}$.
  (3) Implement the OMP algorithm and reconstruct the most-sparse wavelet coefficient ${\hat{W}}_{j}$. These procedures are as follows:
    Step One: initialize residue, $r_{t}{|}_{t=0}=y$, and index set, $A_{t}=\phi$ (empty set);
    For each iteration *t* from 1 to *K* (here, *K* = 8);
    Begin;
    Step Two: the inner product is calculated $\langle r_{t}\Phi \rangle$;
    Then, the column whose inner product is the maximum in Φ is obtained: $\lambda_{t}=arg\underset{t=1\sim N}{\max}\left|\langle r_{t-1}\cdot \Phi_{t}\rangle \right|$; The subscript $A_{t}=\left[A_{t-1},A_{\lambda_{t}}\right]$ is stored, and the most orthogonal column of Φ: $\Phi_{t}=\Phi_{t-1}\cup \left\{\Phi_{\lambda_{t}}\right\}$, the selected column of Φ, is set to **0**;
    Step Three: The least-squares method $\omega_{t}=argmin\|y-\Phi_{t}\omega_{t}{\|}_{2}={\left(\Phi_{t}^{H}\Phi_{t}\right)}^{-1}\Phi_{t}^{H}y$ is implemented;
    Step Four: Approximation $y_{t}=\Phi_{t}\omega_{t}=\Phi_{t}{\left(\Phi_{t}^{H}\Phi_{t}\right)}^{-1}\Phi_{t}^{H}y$ is updated;
    The residue, $r_{t}=y-y_{t}$, is updated;
    End.
  (4) Utilize the inverse wavelet transform for the approximate coefficients ${\hat{W}}_{j}(A_{j})=\omega_{t}$, and the RMF signal is then re-established.
iii If the channel number *i* < 18, return to step iv or end the process.

**Figure 7:**
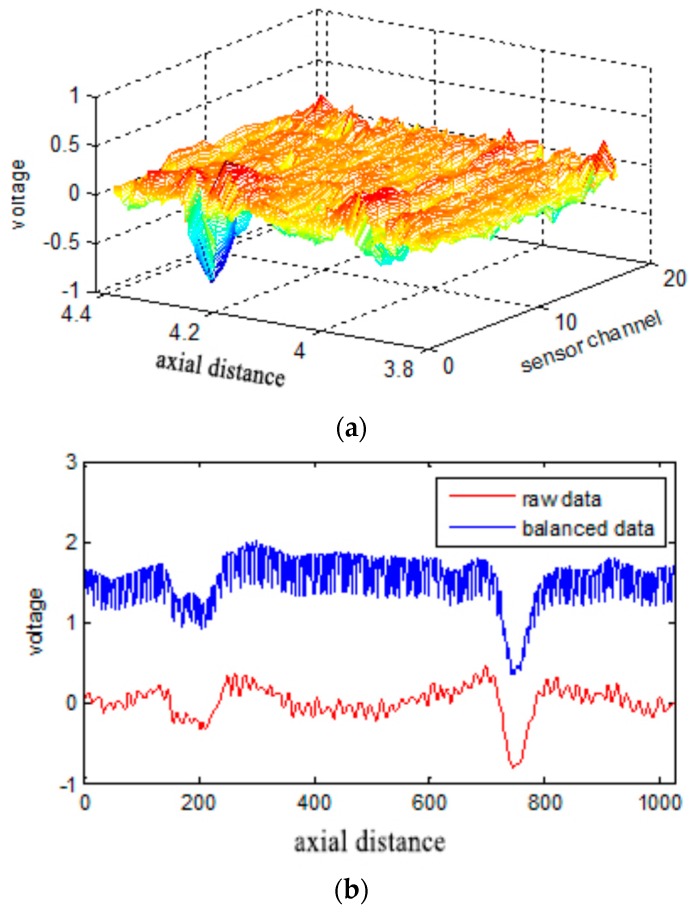
(**a**) The surface chart of the balanced data (the raw data appear in Figure 5b; (**b**) Comparison of a single channel of raw data with the balanced data (the balanced data is obtained from Figure 7a and the raw data from Figure 6a).

The number of IMFs, denoted as *TNM*, obtained by decomposing an RMF signal using the HHT is determined by the expression:

(12) TNM=fix(log(xsize))−1,

where xsize is the length of the signal and fix(·) is the integer operation. Five hundred points were extended to each signal boundary, and, in each channel, the wire rope RMF was sampled for about 13,000 data points. Each signal was decomposed to TNM + 1 IMFs, and the last value is the residue. In this paper, the final component was the direct current component. Implementing the HHT for the other IMFs from the beginning of the NO. 9 component results in the stabilization of the instantaneous frequency of each branch. These branches have illusive components and DC component. Thus, these values were eliminated because they represent useless components of the signal. The balanced RMF data and a comparison with the raw data are presented in Figure 7.

**Figure 8:**
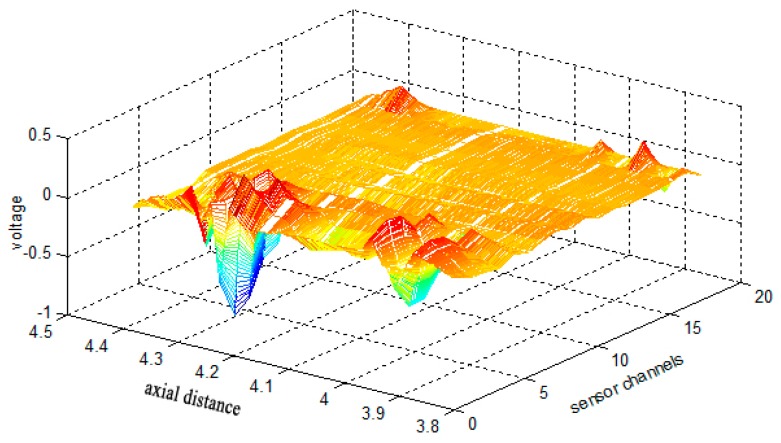
Image of a defect signal with system noise suppressed by means of the proposed HHT and CSWF algorithm (raw data obtained from Figure 5b).

In this paper, CSWF is employed to suppress the high frequency noise and strand waves in the raw data. While the signal is not sparse in the time domain, it is sparse under the wavelet transform. The third Daubechies wavelet was selected to implement the Mallat decomposition algorithm. This type of wavelet has double orthogonality, and its shape is similar to that of the defect signal from contrasting the defect wave. The noise coefficients can be mapped to high frequencies, and the values of the noise coefficients are much smaller than those of the defect coefficients, which is helpful for reconstructing a sparse optimal defect signal by CSWF. For a defect signal of unknown sparsity, employing a slightly larger sparsity (*K* = 20) can ensure an accurate reconstruction. In addition, the majority of high-frequency coefficients were set to 0 so that the recovered signal was clean. Considerable memory and computation time was required for sampling and recovering the long discrete signal; hence, the signal was divided into several segments. The spatial length of each data block was about 2000 sampling points, and the wavelet transform level was 8 because the higher level the wavelet coefficient the more sparse it is. The proposed algorithm was applied to the raw data, and the image of a defect signal obtained from Figure 5b is shown in Figure 8.

## 4. RMF Image Processing

The division of the data into several blocks for conducting CSWF has an effect on the resulting RMF image. If a data block contains no defect signal, the optimal solution provided by the OMP algorithm is only strand waves in a few channels. While these strand waves negatively affect the task of locating defects, the effect of strand waves can be suppressed by image processing. To this end, we implemented a smoothing filter mask using the following process.

### 4.1. Morphological Processing and Defect Location Detection

A binary image of the original RMF image can be acquired by implementing threshold processing, which assigns all pixel values below a threshold to 0, and all others effectively to 1. The application of morphological processing, consisting of expansion and corrosion operations, to the binary image is useful for eliminating or preserving singular points in an image. The expansion operation increases the area of a target region, while the corrosion operation has the opposite effect [32]. Defining a structure mask b and a definition domain $D_{b}$, the expansion operation is written as f⊕b for a binary image f(i,j), and it is defined as:

(13) $$\left(f⊕b)(i,j)=\max\{f(i-i^{\prime },j-j^{\prime })+b(i^{\prime },j^{\prime })|\left(i^{\prime },j^{\prime }\right)\in D_{b}\right\}.$$

This operation is applied to f(i,j) by conducting spatial convolution between f(i,j) and b. The structure mask is rotated and moved over the pixels of f(i,j), adding the elements of the structure mask with the pixels in the overlapping region of f(i,j), and applying that value to the pixel residing at the center of the mask. This operation expands the area of a defect in the binary image. According to the definition of the expanded image, the corrosion operation, written as f⊖b, can be defined as:

(14) $$\left(f⊖b)(i,j)=\min\{f(i+i^{\prime },j+j^{\prime })-b(i^{\prime },j^{\prime })|\left(i^{\prime },j^{\prime }\right)\in D_{b}\right\}.$$

This operation is similar to the expansion operation, except that it generates a center pixel value by subtracting the elements of the structure mask with the pixels in the overlapping region of f(i,j). This operation contracts the area of a defect in the binary image.

**Figure 9:**
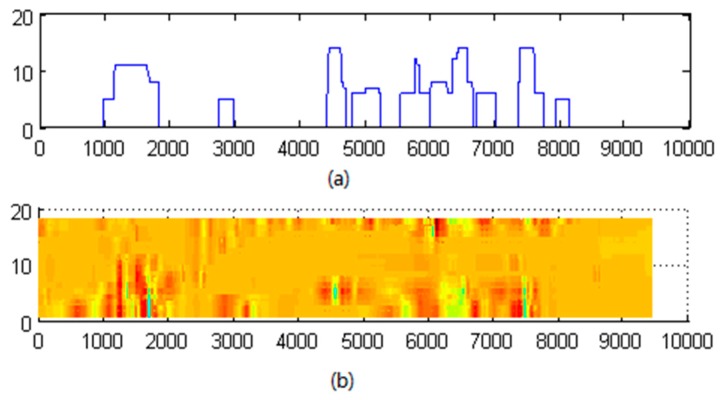
Mapped binary data (**a**) and original filtered wire rope RMF image (**b**), indicating that the mapped data provides the locations of defects.

After morphological processing, the binary image is reserved in these regions and its amplitude is large. We mapped the binary image to the axial direction by summing up the binary values of all 18 channels at each sampling point. This provided an amplitude curve reflecting the axial location of defects. In addition, the defect image was extracted from the wire rope RMF image. Figure 9 presents a comparison of mapped binary data with the original filtered RMF image, which indicates that the mapped data provides the locations of defects in the RMF image.

### 4.2. Normalization and Resolution Enhancement

Morphological processing provides the axial position of defects. Multiplying by filtered image, the binary image transformed an image with clear defects. However, the center of each defect image may not be the minimum of defect, which is detrimental for extracting the characteristics of the defect image. Thus, the center of the defect image must be normalized to the center. In our experiments, the lengths of defects was not more than 2 cm. Theoretically then, the sampling of defects represents 63 points at most. However, the high sensitivity of the GMR sensors will require a much greater number of sampling points to effectively capture a defect. To ensure the acquisition of a complete defect image, 200 sampling points were employed.

The following steps were employed to extract defect locations from a target region:

1. The position $\left(i_{c},j_{c}\right)$ of the minimum of defect is obtained by searching the modulus maximum of a target region image in multiplied image. Then, the axial center is $\left(i_{c},j_{c}\right)$.
2. In the target region, the defect image g can be expressed as:
  (15) $$g(i,j)=\{g(i,j)|i\in [i_{c}-99,i_{c}+100],j_{c}\in [0,N-1]\},$$
  where *N* is the number of channels.
3. If $j_{c}<N/2$, g(i,j) is given as follows:
  (16) $$\{\begin{array}{c} g(i,j+N/2-j_{c})=g(i,j),\;0\leq j\leq N-j_{c}-1\\ g(i,j-N+j_{c})=g(i,j),\;N-j_{c}\leq j\leq N-1 \end{array}\;$$
  If $j_{c}>N/2$, g(i,j) is given as follows:
  (17) $$\{\begin{array}{c} g(i,j+j_{c}-N/2)=g(i,j),\;0\leq j\leq j_{c}-N/2\\ g(i,j-j_{c}+N/2)=g(i,j),\;j_{c}-N/2<j\leq N-1 \end{array}$$

After performing the above operation, the defect image was unrolled as the minimum of the defect RMF image. Due to differences in wire rope diameters, the RMF amplitudes of equivalent broken wires were not the same. To avoid this effect, the amplitude of the defects obtained in a given rope are normalized according to:

(18) g(i,j)=255∗(g(i,j)−gmin)/(gmax−gmin),

where gmin is the minimum amplitude of all the defects in the rope, and gmax is the maximum. For convenient image processing, the values of the defects were normalized to a range of 0–255.

**Figure 10:**
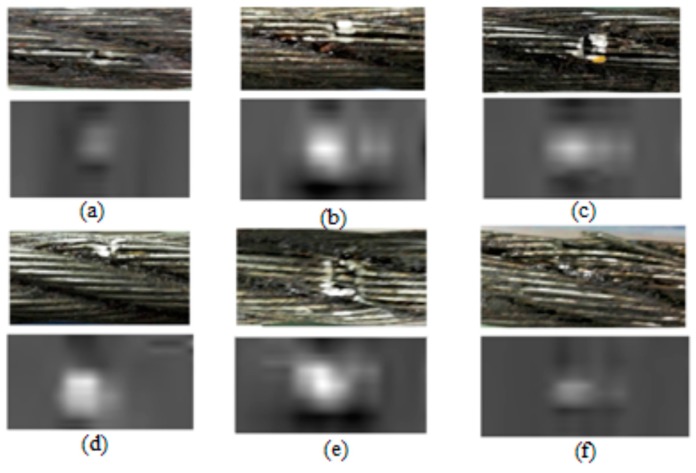
Images of broken wires (top) and high-resolution gray-level RMF images of the corresponding broken wires (bottom): (**a**) one broken wire; (**b**) two broken wires; (**c**) three broken wires; (**d**) five broken wires; (**e**) seven broken wires; (**f**) warping two wires.

In this paper, the diameter of the sensor array was 50 mm and the number of sensors was 18, so the circumferential resolution of the detector was poor, which is detrimental for extracting the characteristics of the image. Therefore, the spline interpolation method was used to improve the resolution by interpolating the number of circumferential points to 200. Figure 10 presents images of several broken wires and their corresponding gray-level RMF images.

## 5. Detection of Broken Wires

### 5.1. Extracting Artificial Image Characteristics

The processing steps discussed above were employed to obtain 200 × 200 pixel gray-level RMF images of defects. Two types of features were selected to extract the characteristics of the images. One feature was the statistical texture, which describes regional characteristics and the basic properties of the image. The other feature was the invariant moment, which describes the gray-level distribution in the region. The invariant moment is calculated using all the pixels in the image, and is not affected by moving or rotating the image [32]. Six statistical texture features and 7 invariant moments were selected as the image characteristics.

The basis of the statistical texture is the histogram of the image, and describing the shape distribution of the histogram relies on the central moment [33], which is defined as:

(19) $$\mu_{n}=\sum_{i=0}^{L-1}{\left(z_{i}-m\right)}^{n}p(z_{i}),$$

where *n* is the order of the moments, $p(z_{i})$ is the normalized histogram, *L* is a random quantity of gray level $z_{i}$, and *m* is the average brightness:

(20) $$m=\sum_{i=0}^{L-1}z_{i}p(z_{i}).$$

The six statistical textures can be defined according to the region brightness features: average brightness (Equation (20)), average contrast, relative smoothness, third-order moment (the skewness of the histogram), conformance, and entropy. The average contrast is defined as:

(21) $$\sigma =\sqrt{\mu_{2}(z)}=\sqrt{\sigma^{2}},$$

which is the standard deviation of the image. It reflects the average change in the image, and the second-order moment is the variance of the image. The relative smoothness is defined as:

(22) $$R=1-1/(1+\sigma^{2}).$$

This value measures the relative smoothness of the brightness in the image. For a constant brightness region, *R* is 0, but, for an offset gray-level image, *R* is 1. The third-order moment is defined as:

(23) $$\mu_{3}=\sum_{i=0}^{L-1}{\left(z_{i}-m\right)}^{3}p(z_{i}).$$

If the histogram is symmetric, *µ*_3_ is zero. If the skewness tends to the right, the value is positive, but, if it tends to the left, the value is negative. The conformance is defined as:

(24) $$U=\sum_{i=0}^{L-1}p^{2}(z_{i}),$$

and, where the image is constant, *U* attains a maximum value. The entropy reflects the degree of randomness in the gray-level values, and is defined as:

(25) $$e=-\sum_{i=0}^{L-1}p(z_{i})\log_{2}p(z_{i}).$$

The invariant moment characteristic is based on a statistical analysis of the gray-level distribution of an image. It is an average statistical description of all the features of the image, and is not easily affected by noise, shifting, rotation, or changes in the size of the image. For an image f(x,y), a varied order exists if it is piecewise continuous, with a limited non-zero number available in the image [34]. The (p+q)-th order moment of f(x,y) is defined as:

(26) $$m_{pq}=\sum_{x}\sum_{y}x^{p}y^{q}f(x,y).$$

The central moments are defined as:

(27) $$u_{pq}={\sum_{x}\sum_{y}\left(x-\bar{x}\right)}^{p}{\left(y-\bar{y}\right)}^{q}f(x,y).$$

where $\bar{x}$ and $\bar{y}$ are the gravity of the image, defined as:

(28) $$\bar{x}=m_{10}/m_{00},\bar{y}=m_{01}/m_{00}.$$

On the basis of the defined central moments, the seven invariant moments are defined as follows [34]:

(29) $$M_{1}=u_{20}+u_{02}$$

(30) $$M_{2}={\left(u_{20}-u_{02}\right)}^{2}+4u_{11}^{2}$$

(31) $$M_{3}={\left(u_{30}-3u_{12}\right)}^{2}+{\left(3u_{21}+u_{03}\right)}^{2}$$

(32) $$M_{4}={\left(u_{30}+u_{12}\right)}^{2}+{\left(u_{21}+u_{03}\right)}^{2}$$

(33) $$\begin{array}{c} M_{5}={\left(u_{30}-3u_{12}\right)}^{2}+(u_{12}+u_{30})[{\left(u_{30}+u_{12}\right)}^{2}-3{\left(u_{21}+u_{03}\right)}^{2}]+\\ (3u_{21}-u_{03})(u_{21}+u_{03})\left[3{\left(u_{30}+u_{12}\right)}^{2}-{\left(u_{21}+u_{03}\right)}^{2}\right] \end{array}$$

(34) $$M_{6}=(u_{20}+u_{02})[{\left(u_{30}+u_{12}\right)}^{2}-{\left(u_{21}+u_{03}\right)}^{2}]+4u_{11}(u_{30}+u_{12})(u_{21}+u_{03})$$

(35) $$\begin{array}{c} M_{7}=\left(3u_{21}-u_{03}\right)\left(u_{30}+u_{12}\right){\left(u_{30}+u_{12}\right)}^{2}-3{\left(u_{21}-u_{03}\right)}^{2}+\\ \left(3u_{12}-u_{30}\right)\left(u_{21}+u_{03}\right)[3{\left(u_{30}+u_{12}\right)}^{2}-{\left(u_{21}+u_{03}\right)}^{2}] \end{array}$$

The seven invariant moments are sensitive characteristics for describing a defect image [22]. The extraction of the characteristics of an image provides an image feature vector. Table 1 lists the components of the image feature vectors obtained for 6 different wire rope samples with 1–5 and 7 broken wires, respectively.

### 5.2. Quantitative Defect Detection

The use of an appropriate classification method is essential for the quantitative inspection of wire rope. The accuracy of defect detection has a direct effect on the accuracy of estimating the residual strength of rope. Thus, the design of the neural network should include good generalization performance and convergence performance, high reliability, and good detection accuracy. The RBF neural network [35] was selected to classify broken wires in this paper according to the characteristics of their defect RMF images. This algorithm represents a forward neural network approach. Its advantages are that any continuous function can be approximated with any degree of accuracy, it is a fast learning approach, it provides a global minimum, and it functions suitably well with classification problems.

The structure of the RBF neural network [36] is similar to that found in multi-layer forward networks, which include input, hidden, and output layers. The input layer consists of the input vectors and a single hidden layer. The number of hidden layers depends on the problem. The map from the input layer to the output layer of the RBF is non-linear, and is seen as a low-dimensional input mapped to a high-dimensional hidden layer with a weight coefficient 1. However, the map from the hidden layer to the output layer is linear, and represents a process of searching the surface of the high dimensional space that fits the data to train the curved surface. The relationship between the input layer and the hidden layer is determined by the center of the activation function. The output of hidden layer is the linear sum of the hidden layer. The RBF neural network adopts a distance function (like the Euclidean distance) or an activation function, such as a Gaussian function. The RBF is radial-symmetric with a center of n-dimensions located far away from the center where the activation degree is low. This property of the hidden layer is denoted as the “local property”.

In experiments, we employed 123 wire rope samples with various instances of broken wires, including 1–5 and 7 broken wires with three different conditions, including wires with a small gap, wires with a wide fracture, and curling wires. To ensure a high generalization performance and recognition accuracy of the neural network, about 75% of the samples (91) were employed to train the network, and the others were used to test its performance. We adopted the MATLAB neural network toolbox to design the RBF classification network, using the tool function newpnn. The designed network consisted of three layers, an input with a number of input vectors and the distance function as the activation function. The hidden layer was a competitive layer without a threshold value, and the input of this layer was the distance between the input vectors and the sample vectors. The probability of each element was calculated through the competitive transfer function. The output of the function was 1 while the maximum probability of the other elements was 0. The “spread, the dispersion parameter determines the smoothness of approximate function” of the RBF was 0.12, and the hidden nodes adopted the automatic optimization strategy, fixing the number of the hidden units to meet the target mean squared error. The activation function of the hidden layer was “complete”. Our network training accuracy was 95.6%, the average training error was 0.0659, and the training time was 0.050212 s. The defect detection accuracy of the trained network was as high as 93.75% when the absolute limiting error for the detected number of broken wires was 2. The average detection error was 0.7813 wires. The network performance is listed in Table 2 for RBFs with different spread values.

**Figure 11:**
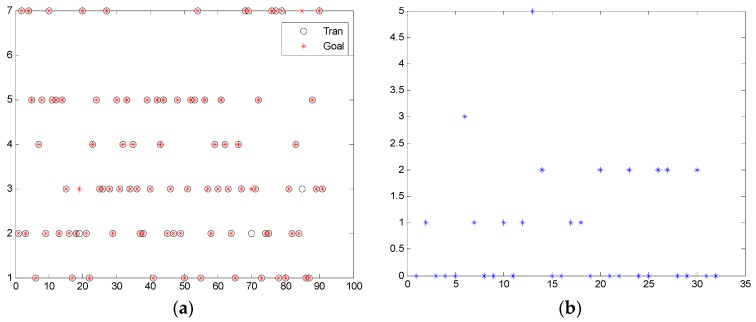
Performance of the designed RBF classification network: (**a**) training performance; (**b**) plot of the detection error.

Figure 11a shows the training results of the designed network. Figure 11b shows the detection error plot. From this figure, it is obvious that the performance of the designed network is good, because the error mainly concentrates on 1 or 2.

## 6. Results and Discussion

RMF images of various wire ropes were acquired using the prototype device, and the above discussed procedures were applied to detect the occurrence and location of wire defects in addition to the number of broken wires included within the defect.

In comparison with previously published results using only wavelet filtering in conjunction with the use of the shape characteristics of the defect RMF image and a BP neural network [22], the novel filtering algorithm proposed in this paper is more adaptive because the HHT is applicable to any unknown signal, while wavelet filtering suffers from the substantial disadvantage regarding an appropriate choice for the wavelet basis. In addition, the statistical textures employed in the present work are more sensitive than the shape characteristics of a defect RMF image. Finally, the RBF neural network also performs better than BP neural network owing to its rapid convergence, global optimization, and good classification performance. The experimental results obtained using the proposed method are therefore more accurate than those obtained by the previously proposed method. The proposed method provided a limiting detection error of 2 wires, where this ranged from 2.565 wires (for a 9 × 19 wire rope) to 3.33 wires (for a 6 × 36 wire rope) in the previous work.

In the experiments, the estimation of the number of broken wires was sensitive to the lift-off distance, where, with different wire rope diameters, samples with an equivalent number of broken wires were not estimated as such, which was detrimental to the subsequent processing. Furthermore, because of the unknown sparsity during the reconstruction of the sparse coefficients, the selection of the optimal solution was either too much or not enough, it is usually less than 20. Thus, the value of *K* must be adjusted when filtering. When designing the classification network, a large spread for the RBF can lead to network non-convergence, while an overly small spread results in network over-fitting, as can be seen from the information given in Table 2.

## 7. Conclusions

A prototype based on the RMF of ferromagnetic materials was designed to detect defects in wire ropes. This inspection system is superior to traditional detection methods in that it offers a high accuracy with a small volume and light weight. The service life of the equipment is longer than that of traditional devices since it suffers from less friction due to the high lift-off distance. The proposed filtering algorithm based on the HHT and CS wavelet filtering suppresses the system noise, balances the data channels, and improves the signal-to-noise ratio. Our experimental results show that the algorithm is good at suppressing the noise in the RMF signal of the wire ropes. We implemented digital image processing to locate and segment the local defects from the rope’s gray image. The statistical texture and invariant moment characteristics were extracted as the input vectors of the RBF neural network, which was used to classify the various broken wires. This recognition system can classify different kind of defects, the successful rate is 93.75% under a limiting error of two wires (the average error is 0.7813). Our future work will focus on training a network with greater accuracy and generalization by extracting additional defect samples and image features. This work has an impact on the determination of the residual strength of a wire rope and the length of its service lifetime.

## Figures and Tables

**Table 1:** Components of the image feature vectors obtained for different wire rope samples.

Number of Broken Wires	1	2	3	4	5	7
m	102	254	231	164	251	174
σ	17.3	4.12	20	19.4	15.4	28.57
R	4.60 × 10^−3^	2.61 × 10^−4^	6.11 × 10^−3^	5.73 × 10^−3^	3.61 × 10^−3^	1.24 × 10^−2^
$\mu_{3}$	−0.076	−0.009	−0.35	−0.112	−0.267	−0.549
U	0.023	0.947	0.071	0.02	0.758	0.068
e	5.91	0.33	4.99	6.1	1.35	5.65
**M1**	1.71 × 10^−3^	5.66 × 10^−4^	7.33 × 10^−4^	1.04 × 10^−3^	6.48 × 10^−4^	1.01 × 10^−3^
**M2**	7.42 × 10^−9^	1.89 × 10^−11^	8.08 × 10^−11^	1.72 × 10^−10^	5.39 × 10^−12^	3.18 × 10^−10^
**M3**	1.82 × 10^−12^	6.70 × 10^−15^	1.04 × 10^−13^	6.27 × 10^−13^	5.68 × 10^−16^	4.22 × 10^−15^
**M4**	2.62 × 10^−12^	8.36 × 10^−15^	6.85 × 10^−14^	1.08 × 10^−12^	5.37 × 10^−15^	1.50 × 10^−13^
**M5**	4.79 × 10^−24^	2.60 × 10^−29^	−5.03 × 10^−27^	8.05 × 10^−25^	9.31 × 10^−3^°	3.09 × 10^−27^
**M6**	1.95 × 10^−15^	−1.14 × 10^−18^	−2.39 × 10^−17^	4.37 × 10^−17^	1.49 × 10^−18^	4.55 × 10^−17^
**M7**	−3.11 × 10^−24^	5.68 × 10^−29^	2.84 × 10^−27^	3.69 × 10^−25^	−9.77 × 10^−31^	−2.19 × 10^−27^

**Table 2:** Performance of the various RBF classification networks when the absolute limiting error was 2 wires.

Spread	Maximum Error	Average Broken Wires Error	Training Accuracy	Recognition
0.05	5	1.25	1	78.13%
0.10	5	1.0313	96.70%	84.38%
0.12	5	0.7813	95.60%	93.75%
0.15	5	1	86.81%	87.50%
